# An Account of a Caries of the Spine, and of the Appearances on Dissection

**Published:** 1781-04

**Authors:** 


II. *An Account of a Caries of the Spine, and of the Appearances on Dissection. By* Samuel Foart Simmons, M. D. F. R. S. *Member of the College of Physicians, London, and of the Royal Medical Society at Paris. Read* April 16, 1781.

THOMAS Mathews of Great Saint Andrew's-ſtreet, Seven-Dials, a thin middle-ſized man, aged 35 years, in 1778 fell backward upon a large table with ſo much violence as to break the frame of the table. No immediate bad conſequence ſucceeded this fall; but about four months afterwards his wife obſerved that his back grew out, and ſoon after this he began to be ſenſible of a weakneſs in his lower limbs, which by degrees became paralytic. About that time he was admitted as a patient under Dr. Hunter, at the Weſtminſter General Diſpenſary, and had a large iſſue made on each ſide of the tumour of the ſpine, according to Mr. Pott's method. The diſcharge was kept open for a conſiderable time, but without benefiting the patient, as the paralytic affection continued to increaſe. He was then admitted into the Middleſex Hoſpital; but to his other complaints there was now added a troubleſome cough, attended tended with a copious purulent expectoration, ſo that he was induced to quit the hoſpital, and apply again for admiſſion at the Diſpenſary. In coughing he ſometimes expectorated portions of bone of the ſize of a pin's head, and ſometimes larger.

He continued nearly in the ſame ſtate, confined to his room and his bed, having little or no uſe of his legs, till Dr. Hunter's departure for Jamaica in December 1780, when he became my patient.

The curvature of the ſpine, which was then conſiderably increaſed, ſeemed chiefly to proceed from the ſeventh dorſal vertebra. He had likewiſe a tumour near the pit of his ſtomach, on the right ſide of the ſternum, which, when he coughed, was much diſtended, ſo as to be as large as a hen's egg, and to the touch ſeemed to contain matter.

The only medicines the patient took were a laxative pill occaſionally to obviate coſtiveneſs, and now and then an opiate to moderate his cough.

About the middle of January 1781, the abſceſs at the pit of his ſtomach burſt, and diſcharged a great quantity of matter. In about a fortnight fortnight after this he began to find his ſtrength abate, and on the 15th of February died. On the 20th, I aſſiſted Mr. Ford, ſurgeon to the Weſtminſter Diſpenſary, in opening the body. The abdominal viſcera afforded no other marks of diſeaſe than a preternatural adheſion of the liver to the peritonæum and diaphragm; the liver itſelf, the ſpleen, ſtomach, inteſtines, and kidneys being apparently ſound. The omentum was much waſted, as might have been expected in a ſubject ſo much emaciated.

On raiſing the ſternum, no appearance of matter was to be ſeen in the thorax, but the lungs were diſcoloured, and univerſally diſeaſed, adhering almoſt every where to the pleura and diaphragm. The heart was ſound, and we found only a ſmall quantity of water in the pericardium.

After removing the heart and lungs, we diſcovered the principal ſeat of the diſeaſe to be in the body of the ſeventh dorſal vertebra, which was in a carious ſtate, having an opening into its cavity large enough to admit a little finger. At the ſide of this hole we found a ſmaller one communicating with a part of the diſeaſed lungs which adhered at this place to the pleura. This

 lateral lateral opening, which was juſt large enough to admit a probe, enabled us to account for the expectoration of bone the patient had been occaſionally ſubject to; but for want of time, we could not ſo ſatisfactorily trace the courſe of the matter diſcharged near the ſternum.

The coats of the medulla ſpinalis were in a great meaſure deſtroyed, and the medulla itſelf was ſurrounded with matter, and ſeemed to be in a ſtate of ſuppuration.

